# Antimicrobial and Antibiofilm Activities of Citrus Water-Extracts Obtained by Microwave-Assisted and Conventional Methods

**DOI:** 10.3390/biomedicines6020070

**Published:** 2018-06-17

**Authors:** Leonardo Caputo, Laura Quintieri, Maria Maddalena Cavalluzzi, Giovanni Lentini, Solomon Habtemariam

**Affiliations:** 1 Institute of Sciences of Food Production (CNR-ISPA) National Council of Research, Via G. Amendola, 122/O, 70126 Bari, Italy; leonardo.caputo@ispa.cnr.it (L.C.); laura.quintieri@ispa.cnr.it (L.Q.); 2 Department of Pharmacy-Drug Sciences, University of Studies of Bari Aldo Moro, Via E. Orabona, 4, 70126 Bari, Italy; mariamaddalena.cavalluzzi@uniba.it (M.M.C.); giovanni.lentini@uniba.it (G.L.); 3 Pharmacognosy Research Laboratories & Herbal Analysis Services, University of Greenwich, Central Avenue, Chatham-Maritime, Kent ME4 4TB, UK

**Keywords:** citron, lemon, orange, solvent-free extraction, pseudomonads, staphylococci, *Escherichia*

## Abstract

Citrus pomace is a huge agro-food industrial waste mostly composed of peels and traditionally used as compost or animal feed. Owing to its high content of compounds beneficial to humans (e.g., flavonoids, phenol-like acids, and terpenoids), citrus waste is increasingly used to produce valuable supplements, fragrance, or antimicrobials. However, such processes require sustainable and efficient extraction strategies by solvent-free techniques for environmentally-friendly good practices. In this work, we evaluated the antimicrobial and antibiofilm activity of water extracts of three citrus peels (orange, lemon, and citron) against ten different sanitary relevant bacteria. Both conventional extraction methods using hot water (HWE) and microwave-assisted extraction (MAE) were used. Even though no extract fully inhibited the growth of the target bacteria, these latter (mostly pseudomonads) showed a significant reduction in biofilm biomass. The most active extracts were obtained from orange and lemon peel by using MAE at 100 °C for 8 min. These results showed that citrus peel water infusions by MAE may reduce biofilm formation possibly enhancing the susceptibility of sanitary-related bacteria to disinfection procedures.

## 1. Introduction

Humans contribute to the microbiome biodiversity by being the main reservoirs and carriers of various microorganisms. As some of these microorganisms may occasionally cause severe infections [1,2], various prophylaxis measures have to be implemented to reduce the microbial load. Of particular significance is an approach to reduce the microbial burden in confined environments such as schools, hospitals, and even general medical devices [3,4,5]. Among the main microorganisms with hygienic-sanitary interest are *Staphylococcus* and *Pseudomonas* which can cause several human diseases. They also associated with an increased resistance to a number of antibiotics. Staphylococci are naturally present as saprophytes on the skin and mucous membranes of mammals and generally only a few species are pathogenic, causing serious infections to humans [6]. The pathogenicity of saprophytic bacteria is due to alteration of the microbiome, as in the case of *Staphylococcus epidermidis* and *S. saprophyticus* which may cause atopic dermatitis [7] and urinary tract infections [8], respectively. Unlike staphylococci, pseudomonads can easily adapt to substrates with poor nutrients (i.e., cosmetics) or grow on materials (i.e., medical devices) in contact with human skin or mucosae [1]. Recently, in addition to the most feared pathogen, *Pseudomonas aeruginosa*, some pseudomonads species (e.g., *P. fluorescens*) which are generally considered non-pathogenic are understood to be opportunistic bacteria as they trigger pathogenesis in debilitated and immunocompromised patients [9].

Most of these pathogenic bacteria form biofilms during their growth through distinct cyclic phases of attachment and adhesion to the substrate, proliferation and maturation, and finally a dispersion phase involving detachment of cells that initiate a new biofilm [10]. Biofilm formation is the cause of increased morbidity and mortality in hospital infections. As it is also closely linked to infection persistence and resistance to common antimicrobial agents, its relevance to disinfection strategies is gaining significance [11,12]. Therefore, there is a growing interest in research directed to the identification of new compounds or innovative control strategies against biofilm formation. Antimicrobial compounds extracted from cheap natural materials (i.e., agro-food industrial waste) through sustainable methodologies have been increasingly considered as new sources to meet this need. Among the known advantages of this strategy is the possibility to hinder microbial growth without the risk of yielding drug-resistant strains [13]. The sustainable utilization of citrus fruit processing waste (mostly peels), which at worldwide level may be estimated in about 14 × 10^6^ tons per year [14], could be a promising strategy in bacterial biofilm control. In fact, citrus waste disposal is not only burdensome for manufacturing factories but constitutes an environmental threat because of the high level of fermentable sugars in citrus pomace [14].

Moreover, the recovery of high value products from citrus waste may increase the economic yield of the citrus processing industries. Recently, *Citrus spp.* extracts showed several biological activities [15,16,17] including antimicrobial effects against pathogenic bacteria and fungi [18]. However, antimicrobial compounds have been recovered by extraction with organic solvents or as essential oils. To avoid the use of organic solvents, the processing of citrus waste for obtaining enriched extracts therefore targeted the water-soluble antimicrobial substances. Innovative “green” strategies (water extraction, supercritical fluids, microwave assisted extraction (MAE)) have, however, been shown to overcome such limitations (i.e., organic solvent utilisation) and provide higher yields and energy savings [19]. Even though solvent-free MAE has been investigated to extract antimicrobial plant compounds [20], very limited studies have been carried out on citrus extracts [13,21]. Furthermore, the extraction of citrus peels by using water or saline solution allowed antimicrobial molecules against oral bacteria to be obtained, such as the glycoside phlorin (3,5-dihydroxyphenyl β-d-glucopyranoside) [22,23] and other flavonoids. To increase the antimicrobial activity of water extracts of citrus peels, the time and temperature of the extraction process should also be carefully considered. On the basis of preliminary studies on antibiofilm activity of some citrus extracts [24,25], further detailed studies must be considered to implement a successful strategy that counteract microbial persistence. On this basis, the present study assessed aqueous extracts obtained from peels of highly widespread citrus fruits (lemon and orange) and citron (generally used in drink and candied fruit manufacturing). The extracts obtained through both prolonged infusion in warm water and MAE at a high temperature were assayed for their antibacterial and antibiofilm activities against saprophytic staphylococci and pseudomonads.

## 2. Experimental Section

### 2.1. Plant Material

Citrons (*Citrus medica* [L].cv. Diamante) were kindly provided by “Consorzio del Cedro di Calabria” Association (Santa Maria del Cedro, Italy); sweet oranges (*C. sinensis* [L.] Osbeck cv. Washington Navel) were donated by the organic farm Gabriella Caruso s.r.l. (Corigliano Calabro, Italy); and lemons (*C. lemon* [L.] Burm cv. Sfusato di Amalfi) were collected in a personal orchard (Caputo L., Cellamare, Italy). After washing twice with distilled water, fruits (*ca.* 2 kg) were dried at room temperature for 1 h and peeled. The recovered peels of each fruit sample were immediately cooled on ice and subsequently freeze-dried. The lyophilized peel samples were finely grounded with Osterizer 12-speed blender (Osteriz, Boca Raton, FL, USA) to obtain a fine powder and stored at −20 °C in air tight bags for further analyses.

### 2.2. Hot Water Extraction (HWE)

HWE was performed as reported by Louche et al. [22] with minor modification. Briefly, 2 g of each lyophilized peel was accurately (±0.01 g) weighed and transferred into 50 mL Falcon™ tubes with screw caps containing 24 mL of pre-heated MilliQ water (Merck Millipore, Darmstadt, Germany). The extraction mixture was refluxed at 50 °C for 24 h in a Thermomixer R (Eppendorf, Westbury, NY, USA) shaking at 400 rpm. At the end of extraction, samples were centrifuged at 13,000 rpm for 15 min followed by each supernatant sterile-filtered and freeze-dried.

### 2.3. Microwave-Assisted Extraction (MAE)

Peel powder (1 g) of each citrus peel was dissolved in 12 mL of MilliQ water and extracted using CEM Discover Microwave (CEM Corporation, Cologno al Serio, Italy) operating at 10 W and under the following three experimental conditions: 80 °C for 20 min (MAE1); 100 °C for 8 min (MAE2); or 100 °C for 20 min (MAE3). For each sample was extracted in duplicate and pooled to obtain a total volume of 24 mL. As with the HWE, samples were centrifuged at 13,000 rpm for 15 min, supernatant sterile-filtered and freeze-dried. All extractions were performed in triplicate.

### 2.4. Yield (%) of Peel Extracts and Preparation for Bioassay

The percent yield of different citrus peel extracts from the HWE and MAE was assessed from comparative freeze-dried extract obtained (in triplicate) with respect to the respective freeze-dried peel as a starting material. The lyophilized extracts were dissolved in one tenth-volume with MilliQ water and centrifuged again. The supernatants were sterile-filtered through 0.22 µm cellulose acetate syringe filters (Whatman Inc., Dassel, Germany) and stored at −20 °C until their use.

### 2.5. Bacteria and Culture Conditions

Target strains used in antimicrobial and antibiofilm assays were obtained from the ISPA-CNR microbial collection stored at −80 °C. Before their use, all strains were freshly cultured overnight under aerobic conditions as shown in Table 1. Growth media were purchased from Thermo Fisher Scientific (Rodano, Italy).

### 2.6. Antimicrobial Assay

Extracts were first assayed for antimicrobial activity against the target strains using the agar disk diffusion assay method according to the EUCAST guidelines [26]. Briefly, a sterile cotton swab soaked with 0.5 McFarland solution of each strain was spread on Petri dishes with Muller Hinton agar (tryptone, 17.5 g/L; beef extract, 2 g/L; soluble starch, 1.5 g/L and Agar, 17 g/L). Then, cellulose disks (Oxoid) soaked with 20 µL of each concentrated extract were placed on inoculated agar surface. Disks containing sterile MilliQ water and 30 µg of chloramphenicol (CHF) were included as negative and positive controls. Plates were incubated at 4 °C until they were ready and transferred to 37 °C for 16–20 h incubation. Diameters (nearest millimeter) of inhibition zones around the assayed disks were measured with a caliper from the back of plate held above a dark background. Subsequently, the extracts without antimicrobial activity were evaluated for their inhibitory effect by studying the microbial growth kinetics of the tested strains. Overnight cultures of each strain were centrifuged at 9000 rpm for 5 min and the pellet was washed twice with sterile saline solution (NaCl 0.95%); after washing steps, cells were resuspended in sterile NaCl solution to reach the concentrations of 8 log colony forming unit (cfu)/mL for *Escherichia coli* and *Pseudomonas* spp. (corresponding to the optical density, OD_600nm_ = 0.3) and 7 log cfu/mL for *Staphylococcus spp.* (OD_600nm_ = 0.147). Then, each culture solution was inoculated at a final concentration of ca. 3 log cfu/mL in 96-well flat-bottomed microtiter plates (Corning™, Corning, NY, USA) filled with the appropriate medium (100 µL; Table 1) supplemented with 20 µL of MilliQ water (control), HWE or MAE extracts. Microtiter plates were incubated at the optimal growth temperature (Table 1) for 24 h and cell growth was determined at 10 min-time intervals by measuring OD at 600 nm using an automated Microplate reader (Varioskan Flash, Thermo Fisher, Milan, Italy). Maximum specific growth rate (*μABS*_max_), lag time (*λABS*), and maximum absorbance (*ABS*_max_) were estimated as described by Dalgaard and Koutsoumanis [27]. All tests were performed in triplicate.

### 2.7. Static Biofilm Formation

Biofilm formation was assayed in 96-well microtiter plates and quantified as described by O’Toole [10]. Briefly, control and treated samples, prepared in triplicates as described above, were incubated at 30 and 37 °C for 48 h. After measuring OD at 600 nm after 24 and 48 h incubation, plates were carefully removed and each well was washed twice with distilled water. The biofilm cells adhering to the bottom and side of each well were then stained with crystal violet (CV; 0.1%, *w*/*v*). After a second washing step, biofilm-associated crystal violet was solubilized with 30% acetic acid (*v*/*v*) and its optical density was measured at 570 nm using a Microplate reader (Varioskan Flash).

### 2.8. Statistical Analysis

A two-way analysis of variance (ANOVA) was conducted with SPSS 20.0 IMB–SPSS statistic software version 20 (IMB corp., Chicago, IL, USA) to examine the effects of citrus peel origin and extraction methods. The relationship between time and temperature levels with the growth kinetic parameters of each treated target strains were assessed for the extracts. The same analysis was performed in order to evaluate the effects on planktonic cell optical density and related biofilm biomass. Multi-comparison analyses among mean values was performed by using Fisher’s least significant difference test at 95% Interval Confidence.

## 3. Results and Discussion

Citrus is an important agricultural crop mainly used in food industries for fresh juice production. The resulting peel and pomaces in citrus processing are by-products that have been used as a source of molasses, pectin, and fragrances [21], and antioxidant and antimicrobial compounds such as phenolic acids and flavonoids [19,28,29]. The conventional extraction of these compounds is usually performed by refluxing peels in large quantities of organic solvents such as ethanol, methanol, ethyl acetate, and acetone. Reports on the phytochemical analyses of these extracts have shown the presence of flavonoids, saponins, tannins, alkaloids, and terpenoids [13]. During the last decade, the extraction of citrus by-products from dried peels/pomace with hot water has increasingly taken hold to allow improved recovery (mostly polyphenols and phenolics) and reduce the extraction time [19,30,31,32,33]. Accordingly, we compared herein the antimicrobial and antibiofilm activity of hot water extracts obtained from three different citrus peels. The extractions were performed by either MAE (at two temperature settings) or the conventional extraction method (HWE). MAE methods are faster than conventional heating processes because microwave energy is delivered efficiently to materials with polar or dipole molecules (such as polyphenols, glycosides, and minerals) through molecular interaction with the electromagnetic field; they also offer a rapid transfer of energy to both solvent and raw plant materials [34]. MAE is not widely used to extract polyphenols from plant tissues in water due to its high dielectric factor and low dispersion coefficient [29,34,35]. Nevertheless, its application under these conditions could favor the extraction of flavonoid glycosides, widespread in citrus peels and previously extracted with prolonged hot infusion [30,32,36].

**Figure 1:**
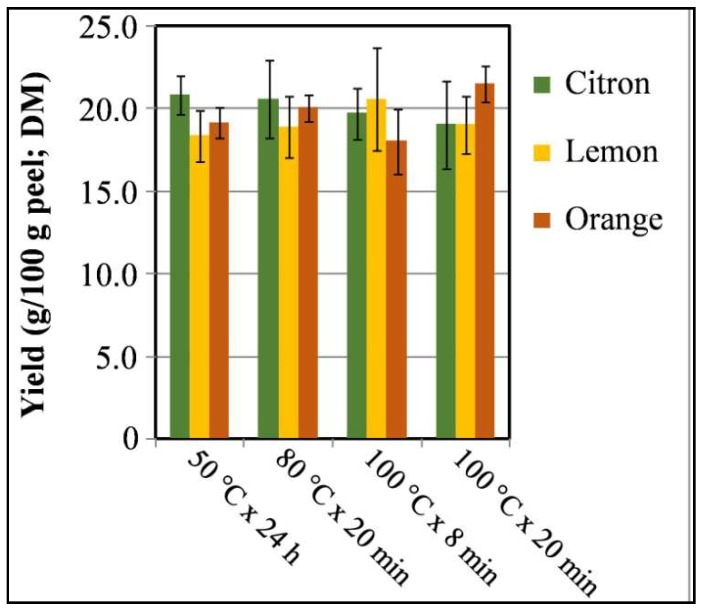
Mean yield percentages (as dry matter) of water extracts obtained from citron, lemon, and orange peels upon hot water extraction (HWE) (50 °C × 24 h) and microwave assisted extraction (MAE) (80 °C × 20 min, 100 °C × 8 min, 100 °C × 20 min). Bars represent standard deviations (*n* = 3).

In the present study, the different extraction methodologies employed did not lead to significant differences in extraction yield in relation to the citrus peel origin or extraction procedures (Figure 1). These values, ranging from 18.0% to 21.5% (as dry matter), were consistent with those previously found by other authors for acetone and ethanol extracts from mandarin peels [37,38] and generally used for other citrus peels. However, concentrated extracts (corresponding to an average concentration of 157 ± 14 µg/mL) showed no inhibitory halos on disc diffusion plates, partially in accordance with data previously reported for aqueous extracts from Brazilian medicinal plants mostly containing tannins [39,40]. Nevertheless, the standardized inhibitory agar diffusion test relies on endpoint growth determination and it is not intended for monitoring microbial kinetics with high temporal resolution in the presence of putative antimicrobial extracts [26]. In fact, inhibitory compounds may exert specific effects on growing microorganisms by impairing cell wall synthesis or substrate uptake and interfering with nucleotides and proteins synthesis [41]. Accordingly, MAE- and HWE-extracts were evaluated for their action on growth kinetics and related primary growth model parameters of target strains in comparison with the untreated control cultures (Table 2 and Table 3). A two-way ANOVA was conducted to examine the effect of three peel origins and five different peel extracts on growth kinetic parameters of several bacterial strains. There was a statistically significant interaction (*p* < 0.001) between the effects of citrus peel origin and extract types on most growth kinetic parameters. In general, simple main effects analysis showed that lemon and orange peel MAE-extracts significantly reduced growth rate and maximum growth levels, and extended lag time of most tested strains compared to the control and HWE-extracts.

**Figure 2:**
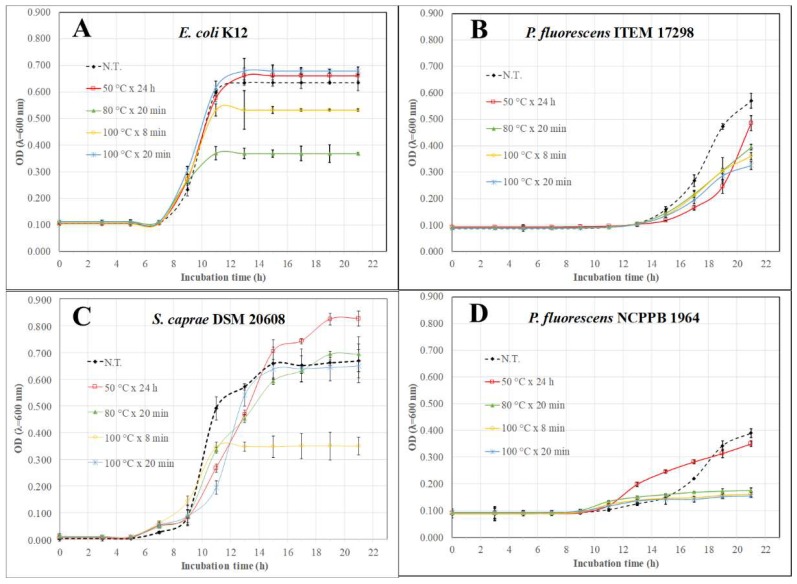
Growth kinetics of some tested strains amended with extracts from citron(**A**), lemon (**B**) and orange (**C**,**D**) peels by hot water extraction (50 °C × 24 h) and microwave assisted extraction (80 °C × 20 min, 100 °C × 8 min, 100 °C × 20 min); N.T.: not treated. Bars represent standard deviation.

For the sake of brevity, only some more significant growth kinetics have been reported for up to 21 h of incubation in Figure 2. Overall, despite the lush growth of untreated cultures, MAE- and HWE-extracts exerted an inhibiting action on target strains resulting in a significant (*p* < 0.01) increase of lag time (*λABS*) and reduction of *ABS*_max_ (Table 2 and Figure 2). As shown in Figure 2A, citron peel MAE-extracts (80 °C × 20 min and 100 °C × 8 min) reduced the maximum *E. coli* K12 turbidity levels by an average of 0.180 OD units compared to the remaining samples even though their lag times were similar (Table 3). All lemon peel extracts were active in efficiently controlling growth rate and the final microbial turbidity of psychrotrophic *P. fluorescens* ITEM 17298 (Figure 2B). While all MAE-extracts from orange peels efficiently kept *P. fluorescens* ITEM 17298 growth very low (Figure 2D), only extracts processed by MAE at 100 °C for 8 min were significantly (*p* < 0.001) effective in reducing the final optical density of *S. caprae* (Figure 2C). Hence, the optimum condition to extract growth modulators would be to lower the temperature to 80 °C and increase extraction time to 20 min.

The primary growth model parameters are generally associated with bacterial adaptation to growth environment, antimicrobial sub-lethal concentrations, and nutrient depletion or toxic metabolite accumulation, respectively [42,43]. In our work, some target strains (*P. fluorescens* 84094, *P. fluorescens* NCPPB 1964, *S. saprophyticus* UR18), mostly growing in the presence of MAE extracts, showed a sharp λ*ABS* increase followed by a relevant *ABS*_max_ decrease. Conversely, at the same conditions, other strains (*P. fluorescens* ITEM 17298 and ITEM 17299, *P. fluorescens* NCPPB 1964) recorded a marked delayed growth and higher final turbidity increases than the related untreated control cultures. This biphasic growth pattern (hormetic effect) resembled that found in bacteria treated with sub-lethal concentrations of antibiotics generally leading to selective enrichment and overgrowth of tolerant bacteria and enhancing dissemination of multidrug resistance [44]. On the other hand, an evident inhibitory effect was recorded for the cultures of the strains ITEM 17298, NCPPB 1964, and UR18 grown in the presence of MAE-lemon and -orange extracts that displayed a significant extension of lag time as well as a reduction of growth rate in comparison with untreated control cultures. Similar effects were also reported for registered water extracts (mostly containing flavonoid glycosides, terpenoids, and phenolic acids) from both *Stevia rebaudiana* and citrus extracts against *Listeria innocua* and different spoilage bacteria and yeasts, respectively [45,46].

**Figure 3:**
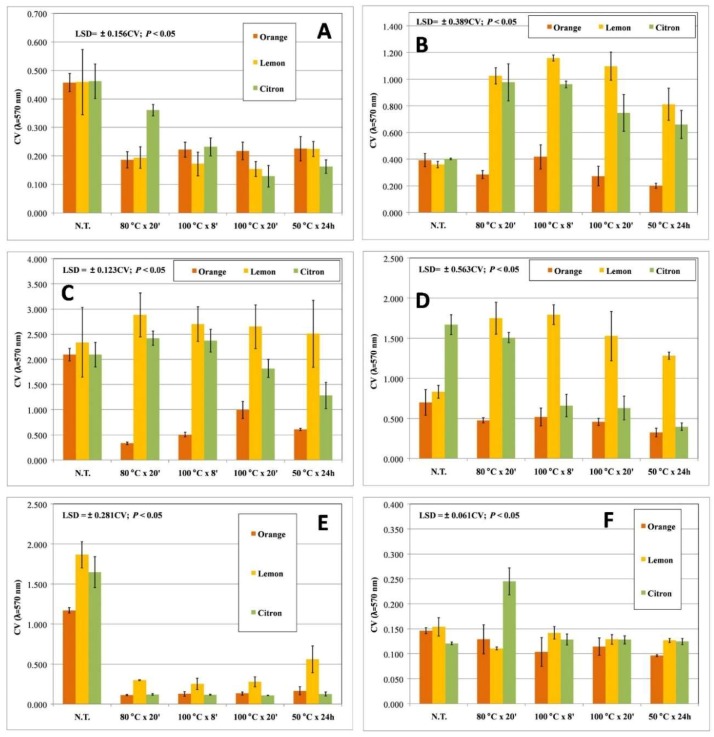
Biofilm biomass produced by *P. fluorescens* NCPPB 1964 (**A**), *P. fluorescens* 84094 (**B**), *P. fluorescens* ITEM 17298 (**C**), *P. fluorescens* ITEM 17299 (**D**), *P. putida* ITEM 17297 (**E**), and *E. coli* K12 (**F**) in the presence of different citrus peel water extracts. Mean values (bars ± standard deviation) from the same strain were compared by Fisher’s least significant difference (LSD) multiple-comparison test (95% Confidence Intervals); N.T.: not treated.

**Figure 4:**
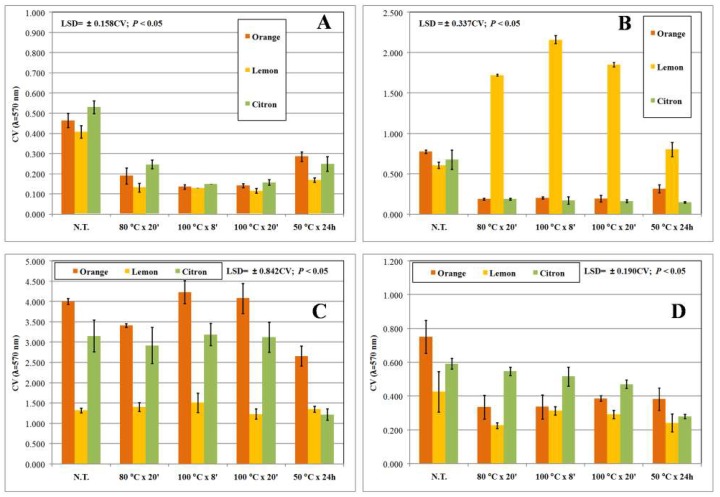
Biofilm biomass produced by *S. caprae* DSM 20608 (**A**), *S. epidermidis* UR63 (**B**), *S. saprophyticus* UR18 (**C**), and *S. xylosus* DSM 20266T (**D**) in the presence of different citrus peel water extracts. Mean values (bars ± standard deviation) from the same strain were compared by Fisher’s least significant difference (LSD) multiple-comparison test (95% Confidence Intervals); N.T.: not treated.

As widely reported, pseudomonads and staphylococci are able to form biofilm on biotic and abiotic surfaces. Biofilm phenotypes are resistant to common control strategies and consequently showed an increased persistence in the environment. In order to find novel antibiofilm agents, each extract was then assayed for their ability to counteract biofilm formation. Overall, untreated cultures produced high amounts of biofilm biomass (0.4 < OD_570nm_ < 4), except for *E. coli* that produced a low amount of biofilm (OD_570nm_ < 0.150; Figure 3F). Two-way ANOVA analysis highlighted that the interaction between the experimental factors, plant origin of the peels, and the extraction method applied, significantly (*p* < 0.001) affected the biofilm biomass of all tested strains except for *E. coli* K12 and *S. saprophyticus* UR18 strains. By contrast, biofilm formation by NCPPB 1964, ITEM 17297, and DSM 20608 strains were reduced regardless of the assayed extract (Figure 3 and Figure 4). The combined treatment (peel origin × extraction procedure) resulted in higher than 50% reduction on only five tested strains that are also considered strong biofilm producers (Figure 3 and Figure 4). Indeed, all extracts fully counteracted ITEM 17297 biofilm formation, whereas orange peel MAE-extracts and citron peel HWE-extracts were similarly active against *P. fluorescens* ITEM 17298, *S. epidermidis* UR63, and *S. saprophyticus* UR18, respectively. Comparable biofilm reduction values were registered only in the cultures of the strains ITEM 17298, ITEM 17299, UR63, and UR18 treated with the citron peel HWE-extracts; by contrast citron extract obtained at 100 °C by MAE was highly active against ITEM 17299 and UR63 (Figure 3 and Figure 4).

These results were partially consistent with those observed on growth patterns described in the first 21 h of incubation. Indeed, it is noteworthy that biofilm formation of the strain ITEM 17297 was fully inhibited by all extracts among which those from lemons affected bacterial lag phase and growth rate (Table 2 and Table 3, and Figure 3). On the other hand, NCPPB 1964 displaying a significant extension of lag time as well as a reduction of growth rate in the presence of lemon and orange MAE-extracts (Table 2 and Table 3, and Figure 2D) showed a scarce but significant (*p* < 0.05) inhibition of biofilm formation (25% on average; Figure 3A). In addition, even though DSM 20608 biofilm was moderately inhibited by all extracts (by about 29%), its growth rate and final growth level were negatively affected only by lemon peel extracts (80 °C × 20 min and 100 °C × 8 min) and orange peel extract (100 °C × 8 min), respectively (Figure 2C and Table 3). Likewise, in the presence of citron peel HWE-extract and orange and lemon peel MAE-extracts ITEM 17298 showed a sharp reduction in growth rate compared to not treated control culture (Table 3 and Figure 2B), whereas biofilm biomass was highly lowered by all orange extracts (Figure 3C). Conversely, all lemon extracts counteracting the growth of the same strain did not show any inhibitory effect on its biofilm formation. Interestingly, citron extracts negatively affected both growth rate and biofilm formation of *S. epidermidis* UR63 (Table 3 and Figure 4B). Even though all tested citrus peel water extracts had no bactericidal effect against several strains, some of them (mostly MAE-extracts) can control bacterial growth parameters and negatively affect biofilm formation of the susceptible strains. Several studies have shown that plant-derived compounds and antimicrobials at sub-lethal doses impair adhesion and bacterial biofilm formation while inducing planktonic growth of a hypermotile phenotype [47,48,49]. This strategy can be used to disarm microorganisms of sanitary interest without killing them or opposing a selective pressure on them but reducing their virulence or increasing susceptibility [50]. Likewise, polyphenols have received some attention regarding their antimicrobial effect upon biofilms produced by *S. epidermidis* [31]; tannins from water-extracts of some Brazilian medicinal plants allowed a bacteriostatic behaviour of pathogen *Pseudomonas aeruginosa* but strongly reduced its biofilm biomass [40]. Considering coumarins, Villa and Cappitelli [51] have recently proposed them as alternative therapeutic strategies based on their ability to block the quorum sensing signalling systems and to inhibit the formation of biofilms in clinically relevant pathogens. Similar evidence was also found for flavonoids abundant in citrus peels, like naringenin against *Streptococcus mutans* and phenolic acids like cinnamic acid against drug multi-resistant *P. aeruginosa* PAO1 [52].

## 4. Conclusions

In this study, aqueous extracts obtained from lemon, orange, and citron peels by either HWE or three MAE procedures were assayed for their antimicrobial and antibiofilm activities against several human skin commensal bacteria and opportunistic pathogens correlated with nosocomial infections. Even though bactericidal effects were not observed, citrus water extracts showed inhibitory activity by negatively affecting lag time, growth rate, and final growth level of most target strains. Compared to conventional HWE, the application of MAE performed at 100 °C significantly affect the number of strains susceptible mostly to orange and lemon citrus peel extracts by significantly reducing growth rate and lag phase. Nevertheless, only some treated susceptible strains (i.e., ITEM 17297 and UR63) formed significantly lesser biofilm amounts than those found in untreated control cultures. Importantly, very high reductions in biofilm biomass were registered in bacterial cultures with at least one growth parameter impaired by the citrus peel extracts. These results, beyond suggesting a possible methodology to screen high numbers of extracts using turbidimetry data, pave the way for a sustainable usage of citrus peel extracts to hinder bacterial biofilm formation.

## Figures and Tables

**Table 1:** Target strains, growth media and culture conditions.

Target Strain	Growth Conditions
*Staphylococcus epidermidis* UR63	Tryptic Soy Broth (TSB), 37 °C, 130 rpm
*Staphylococcus saprophyticus* UR18	
*Staphylococcus caprae* DSM 20608	
*Staphylococcus xylosus* DSM20266T	
*Pseudomonas fluorescens* NCPPB1964	Luria Bertani (LB), 30 °C, 130 rpm
*Pseudomonas fluorescens* ITEM 17298	
*Pseudomonas fluorescens* ITEM 17299	
*Pseudomonas fluorescens* ITEM 84094	
*Pseudomonas putida* ITEM 17297	
*Escherichia coli* K12	Luria Bertani (LB), 37 °C, 130 rpm

**Table 2:** Lag phase (*λABS*) and maximum optical density (*ABS*_max_) shown by bacterial cultures amended with different aqueous extracts of citron, lemon, and orange peels.

Bacterial Strain		Citron	Lemon	Orange	LSD	Citron	Lemon	Orange	LSD
	Extracts	*λABS* (h)				*ABS*_max_			
*E. coli* K12	N.T.	7.4	7.3	6.9	n.s.	0.711	0.699	0.664	0.109
	HWE	9.1	8.6	7.7		0.642	0.785	1.249	
	MAE1	10.1	10.8	9.5		0.368	0.733	1.186	
	MAE2	9.4	9.7	8.9		0.532	0.800	1.000	
	MAE3	9.1	11.1	13.8		0.679	0.787	1.180	
*P. fluorescens* 84094	N.T.	12.3	12.1	11.5	1.9	0.687	0.676	0.642	0.180
	HWE	13.4	7.9	13.8		0.805	0.999	0.611	
	MAE1	12.0	7.6	12.1		1.141	1.036	0.929	
	MAE2	13.8	6.8	12.4		0.747	1.042	0.844	
	MAE3	14.2	8.1	12.2		0.654	0.966	0.909	
*P. fluorescens* ITEM 17298	N.T.	13.5	13.2	12.6	1.5	0.541	0.532	0.506	0.057
	HWE	13.8	13.0	10.8		0.678	0.475	0.549	
	MAE1	15.5	14.3	10.8		0.900	0.298	0.695	
	MAE2	15.6	15.5	10.7		0.735	0.269	0.559	
	MAE3	14.9	14.7	10.7		0.800	0.291	0.523	
*P. fluorescens* ITEM 17299	N.T.	13.1	12.8	12.2	1.6	0.549	0.540	0.513	0.189
	HWE	14.8	14.0	9.7		1.091	1.174	0.772	
	MAE1	14.3	14.7	9.8		1.010	0.897	0.557	
	MAE2	14.3	13.7	11.1		0.956	0.982	0.619	
	MAE3	15.2	14.2	11.5		0.934	0.823	0.524	
*P. fluorescens* NCPPB 1964	N.T.	13.5	13.2	12.6	2.0	0.41	0.41	0.39	0.034
	HWE	20.0	14.0	10.3		0.304	0.516	0.350	
	MAE1	20.0	15.2	21.6		0.293	0.430	0.176	
	MAE2	19.4	14.9	25.5		0.304	0.427	0.161	
	MAE3	16.9	15.0	31.5		0.475	0.420	0.155	
*P. putida* ITEM 17297	N.T.	14.9	14.6	14.1	3.2	0.399	0.392	0.372	0.576
	HWE	16.3	9.8	11.9		0.846	0.994	1.528	
	MAE1	16.5	9.2	13.9		1.592	0.780	1.414	
	MAE2	16.4	7.8	13.6		1.569	0.877	1.426	
	MAE3	16.5	5.2	13.2		1.586	0.930	1.402	
*S. caprae* DSM 20608	N.T.	14.0	13.8	13.1	1.6	0.713	0.700	0.666	0.210
	HWE	13.6	7.3	2.4		1.198	1.293	0.824	
	MAE1	13.3	4.9	8.1		1.108	1.153	0.693	
	MAE2	13.1	5.1	9.3		1.163	1.229	0.349	
	MAE3	13.1	5.7	8.6		1.255	1.206	0.648	
*S. epidermidis* UR63	N.T.	9.3	-	-	1.0	0.902	-	-	0.125
	HWE	8.3	-	-		1.224	-	-	
	MAE1	7.0	-	-		1.248	-	-	
	MAE2	6.6	-	-		1.253	-	-	
	MAE3	7.8	-	-		1.217	-	-	
*S. saprophyticus* UR18	N.T.	-	7.0	6.9	1.3	-	0.354	0.348	0.038
	HWE	-	11.1	9.5		-	0.362	0.342	
	MAE1	-	9.3	10.8		-	0.406	0.400	
	MAE2	-	10.9	10.5		-	0.426	0.318	
	MAE3	-	9.8	14.2		-	0.420	0.414	
*S. xylosus* DSM20266T	N.T.	11.3	11.1	-	1.2	0.901	0.885	-	0.088
	HWE	13.4	3.9	-		1.079	1.063	-	
	MAE1	12.2	8.8	-		1.108	1.041	-	
	MAE2	12.2	9.0	-		1.199	0.430	-	
	MAE3	12.2	8.0	-		1.231	0.807	-	

N.T.: Not treated; HWE: Extract obtained at 50 °C × 24 h; MAE1: Extract obtained at 80 °C × 20 min; MAE2: Extract obtained at 100 °C × 8 min; MAE3: Extract obtained at 100 °C × 20 min. Mean values from the same strain were compared by Fisher’s least significant difference (LSD) multiple-comparison test (95% Confidence Intervals).

**Table 3:** Growth rates (*µABS*) and duplication time (DT) shown by bacterial cultures amended with different aqueous extracts of citron, lemon, and orange peels.

Header		Citron	Lemon	Orange	LSD	Citron	Lemon	Orange	LSD
	Extracts	*µABS* (h^−1^)				DT (h)			
*E. coli* K12	N.T.	0.231	0.227	0.216	0.081	4.3	4.2	4.0	1.9
	HWE	0.420	0.123	0.206		1.6	5.8	3.5	
	MAE1	0.294	0.160	0.220		2.3	4.1	3.3	
	MAE2	0.402	0.220	0.212		1.7	3.5	3.4	
	MAE3	0.426	0.173	0.394		1.6	3.9	1.8	
*P. fluorescens* 84094	N.T.	0.234	0.230	0.219	0.023	3.3	3.2	3.0	0.5
	HWE	0.230	0.166	0.250		2.9	4.3	2.9	
	MAE1	0.225	0.168	0.254		3.0	4.3	2.8	
	MAE2	0.214	0.163	0.256		3.1	4.1	2.8	
	MAE3	0.202	0.165	0.261		3.3	4.0	2.7	
*P. fluorescens* ITEM 17298	N.T.	0.215	0.211	0.201	0.023	3.7	3.6	3.4	0.5
	HWE	0.184	0.192	0.167		3.9	3.5	4.3	
	MAE1	0.309	0.141	0.204		2.3	4.9	3.5	
	MAE2	0.277	0.135	0.174		2.6	5.0	4.1	
	MAE3	0.258	0.139	0.169		2.8	5.0	4.2	
*P. fluorescens* ITEM 17299	N.T.	0.213	0.209	0.199	0.056	3.6	3.5	3.3	0.7
	HWE	0.313	0.331	0.194		2.3	2.2	3.7	
	MAE1	0.289	0.314	0.155		2.5	2.3	4.6	
	MAE2	0.302	0.315	0.194		2.4	2.1	3.7	
	MAE3	0.293	0.286	0.182		2.4	2.4	3.9	
*P. fluorescens* NCPPB 1964	N.T.	0.162	0.159	0.152	0.015	6.5	3.9	6.3	1.1
	HWE	0.113	0.176	0.110		5.6	4.0	12.3	
	MAE1	0.128	0.164	0.056		7.0	4.2	14.1	
	MAE2	0.106	0.161	0.049		3.3	4.2	16.4	
	MAE3	0.215	0.159	0.042		3.8	3.8	3.6	
*P. putida* ITEM 17297	N.T.	0.212	0.208	0.198	0.071	2.0	3.3	2.1	1.3
	HWE	0.351	0.219	0.344		1.4	4.0	1.6	
	MAE1	0.514	0.172	0.438		1.4	4.2	1.7	
	MAE2	0.513	0.163	0.422		1.4	5.2	1.8	
	MAE3	0.528	0.141	0.392		2.1	2.0	1.9	
*S. caprae* DSM 20608	N.T.	0.378	0.372	0.353	0.060	2.1	1.9	1.9	0.3
	HWE	0.334	0.375	0.349		2.4	2.4	1.7	
	MAE1	0.305	0.277	0.399		2.3	2.3	1.9	
	MAE2	0.306	0.292	0.359		2.3	2.1	1.9	
	MAE3	0.315	0.315	0.402		1.3	-		
*S. epidermidis* UR63	N.T.	0.549	-	-	0.057	1.5	-	-	0.4
	HWE	0.494	-	-		1.7	-	-	
	MAE1	0.414	-	-		1.7	-	-	
	MAE2	0.419	-	-		2.0	-	-	
	MAE3	0.352	-	-		-	-	-	
*S. saprophyticus* UR18	N.T.	-	0.155	0.153	0.025	-	4.8	4.7	0.5
	HWE	-	0.169	0.206		-	4.2	3.5	
	MAE1	-	0.164	0.220		-	4.4	3.3	
	MAE2	-	0.148	0.212		-	4.9	3.4	
	MAE3	-	0.163	0.394		-	4.4	1.8	
*S. xylosus* DSM20266T	N.T.	0.341	0.336	-	0.032	2.1	2.0	-	0.30.5
	HWE	0.234	0.332	-		3.1	2.0	-	
	MAE1	0.203	0.407	-		-	4.8	4.7	
	MAE2	0.220	0.272	-		-	4.2	3.5	
	MAE3	0.221	0.274	-		-	4.4	3.3	

N.T.: Not treated; HWE: Extract obtained at 50 °C × 24 h; MAE1: Extract obtained at 80 °C × 20 min; MAE2: Extract obtained at 100 °C × 8 min; MAE3: Extract obtained at 100 °C × 20 min. Mean values from the same strain were compared by Fisher’s least significant difference (LSD) multiple-comparison test (95% Confidence Intervals).

## Abbreviations

*ABS_max_*	Maximum absorbance
DT	Duplication time
HWE	Hot water extraction
*λABS*	Lag time
LSD	Least significant difference
HWE	Hot water extraction
MAE	Microwave-assisted extraction
*μABS*_max_	Maximum specific growth rate
N.T.	Not treated
CFH	Chloramphenicol
